# Remission of Severe, Relapsed, and Refractory TTP after Multiple Cycles of Bortezomib

**DOI:** 10.1155/2017/9681832

**Published:** 2017-03-06

**Authors:** Manu R. Pandey, Pankit Vachhani, Evelena P. Ontiveros

**Affiliations:** ^1^Department of Internal Medicine, Jacobs School of Medicine and Biomedical Sciences, Buffalo, NY, USA; ^2^Leukemia Service, Department of Medicine, Roswell Park Cancer Institute, Buffalo, NY, USA

## Abstract

Acquired thrombotic thrombocytopenic purpura (TTP) is characterized by autoantibodies against a disintegrin and metalloproteinase with a thrombospondin type 1 motif, member 13 (ADAMTS13). Uncleaved von Willebrand factor (VWF) multimers accumulate and bind to platelets which causes spontaneous microthrombi ultimately causing microangiopathic hemolytic anemia, thrombocytopenia, and end-organ ischemia. Plasma exchange (PEX) with or without steroids constitutes standard first-line therapy with rituximab typically reserved for refractory cases. Therapies beyond rituximab lack strong evidence for routine use. Recently, bortezomib, a proteasome inhibitor used commonly in patients with multiple myeloma, was shown to induce remission in patients with refractory TTP. Here, we report a case of severe, relapsed TTP that was refractory to PEX, steroids, and rituximab that underwent remission following three cycles of bortezomib. We discuss the salient features of our case, the mechanism of action of bortezomib, and the very few other similar reports that exist in the literature. We conclude that bortezomib should be considered for patients with TTP refractory to PEX, steroids, and rituximab due to its efficacy and relatively favorable side effect profile.

## 1. Introduction

Thrombotic thrombocytopenic purpura (TTP) is a rare disorder with an estimated annual incidence of 2.9 cases per million in the US [1]. An individual patient, though, can have multiple relapses over many years. Classically, TTP presents with fever, neurologic deficits, thrombocytopenia, microangiopathic hemolytic anemia (MAHA), and renal failure [2]. However, this pentad is extremely rare (<5% of presentation), partly due to early recognition and treatment of the condition, and only MAHA and thrombocytopenia are the two requisite features for TTP. Acquired TTP occurs due to the deficiency of von Willebrand factor (VWF) cleaving protein, also known as a disintegrin and metalloproteinase with a thrombospondin type 1 motif, member 13 (ADAMTS13) due to anti-ADAMTS13 autoantibodies [2]. Absence of ADAMTS13 activity leads to accumulation of uncleaved ultra-large VWF multimers which bind to platelets and cause spontaneous microthrombi in conditions of high shear environments, thus causing MAHA and microvascular occlusion which results in end-organ ischemia [2].

Rapid initiation of plasma exchange (PEX) and immunosuppressive therapy (corticosteroids typically) constitutes the standard-of-care for acquired TTP [2]. Rituximab, as an adjunct to PEX with or without corticosteroids, has emerged as an option for patients with relapsed/refractory TTP; response rates in excess of 80% have been observed in various small studies [3–5]. Other second-line therapies include cyclosporine, cyclophosphamide, vincristine, and splenectomy with lower rates of remission and long-term side effects [2]. Recently, a few cases of bortezomib (Velcade®, Millennium Pharmaceuticals Inc., Cambridge, MA, USA) induced remission of TTP have emerged [6–12]. However, considerable heterogeneity exists among these cases.

Here, we describe the report of a young woman with severe, relapsed, and refractory TTP who subsequently achieved remission with three cycles of bortezomib.

## 2. Case Presentation

**Figure 1:**
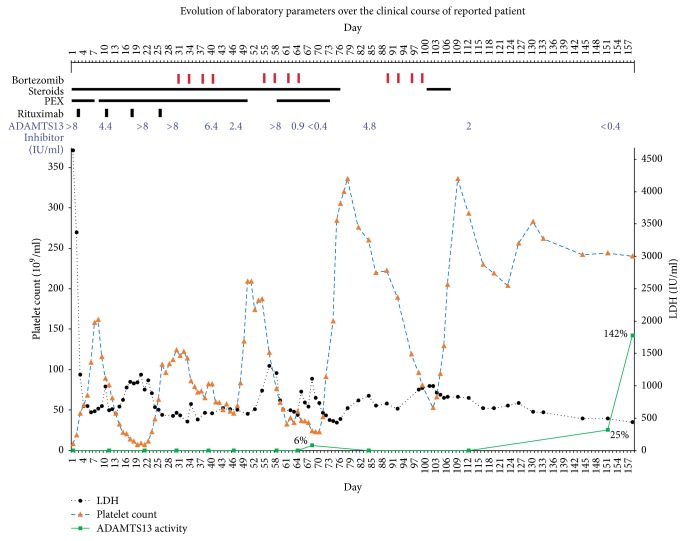
Evolution of laboratory parameters demonstrating patient's clinical course during the relapsed TTP episode. Major therapies delivered have been indicated. LDH: lactate dehydrogenase; PEX: plasma exchange.

A 33-year-old Caucasian woman with no significant medical history was diagnosed with TTP in 2009. She was successfully treated with daily PEX and four doses of weekly rituximab. The disease relapsed in 2012 and was again managed with PEX and rituximab. In 2015 she presented with nonbloody emesis and right flank pain associated with urinary tract infection. Key laboratory findings demonstrating the clinical course are shown in Figure 1. Complete blood count (CBC) revealed leukocytes 11,000/*μ*L (4,000–11,000/*μ*L), hemoglobin 10.8 g/dL (12.5–15.5 g/dL), and platelets 8,000/*μ*L (150,000–450,000/*μ*L). Other notable laboratory findings included negative direct Coombs test, lactate dehydrogenase (LDH) of 4636 IU/L (313–618 IU/L), serum creatinine of 3.25 mg/dL (0.52–1.04 mg/dL), ADAMTS13 activity level of <5% as determined by FRETS-VWF73 substrate (reference range: ≥67%), and ADAMTS13 inhibitor level > 8 inhibitor units/mL (IU/mL; reference range: ≤0.4 IU/mL). Diagnosis of a second relapse of TTP was made. Treatment with PEX and high-dose steroids daily was started (day 1). Rituximab 375 mg/m^2^ was administered on a weekly basis starting day 3. Platelet count increased to more than 150,000/*μ*L for two consecutive days by day 7. PEX was held on day 8 and platelet count dropped to 89,000/*μ*L the next day. PEX was restarted while continuing daily steroid and weekly rituximab. Thrombocytopenia initially worsened with the platelet count reaching a nadir of 7,000/*μ*L on day 19 and then improved to a peak of 124,000/*μ*L by day 30. However, ADAMTS13 activity <5% and ADAMTS13 inhibitor > 8 IU/mL persisted (days 21, 30). Flow cytometry based quantification of peripheral blood CD19 or CD20 positive B-cell lymphocytes was not performed to establish or refute efficacy of rituximab. However, given the ADAMTS13 laboratory profile, the patient's TTP was considered refractory to PEX, steroids, and rituximab (total four infusions). The latter was then discontinued. Due to refractory TTP and emergence of few case reports of successful use of bortezomib in this setting, cycle 1 of off-label bortezomib 1.3 mg/m^2^ was administered intravenously on days 30, 33, 37, and 40 along with plasma exchange and steroids. While ADAMTS13 inhibitor level decreased to 2.4 IU/mL, ADAMTS13 activity level < 5% persisted on day 46. LDH had trended down to 633 IU/L by day 50. Patient was discharged with platelet count of 185,000/*μ*L on day 52. By day 58 platelet count decreased to 76,000/*μ*L while LDH increased to 1193 IU/L and she was readmitted. Additionally, ADAMTS13 activity level was <5% with ADAMTS13 inhibitor levels increased again to >8 IU/mL. PEX was restarted while high-dose steroids were continued. Due to refractory disease, cycle 2 of bortezomib 1.3 mg/m^2^ was administered subcutaneously on days 55, 58, 62, and 65. She developed mild peripheral neuropathy that was attributed to bortezomib. ADAMTS13 activity level improved to 6% and ADAMTS13 inhibitor level improved to <0.4 IU/mL by day 68. Platelet count increased to 320,000/*μ*L and LDH improved to 405 by day 77. By day 82, though, worsening laboratory parameters were noted: LDH of 846 IU/L, platelet count of 260,000/*μ*L, ADAMTS13 inhibitor of 4.8 IU/mL, and ADAMTS13 level of <5%. As such, cycle 3 of bortezomib was administered subcutaneously on days 89, 92, 96, and 99. PEX was offered because LDH increased to 996 IU/L on day 100 and platelet count decreased to 53,000/*μ*L on day 102, but patient refused. High-dose steroid was added for five days. Platelet counts recovered by day 106. ADAMTS13 activity increased to 8% and the ADAMTS13 inhibitor decreased to 2 IU/mL by day 112. By day 151, ADAMTS13 activity increased further to 25% and ADAMTS13 inhibitor decreased to <0.4 IU/mL. Patient continues to be in remission since then. On last check, ADAMTS13 activity was 142% and ADAMTS13 inhibitor was undetectable on day 315; LDH was 393 IU/L, platelets were 247,000/*μ*L, and serum creatinine was 0.72 mg/dL on day 543. She continues to follow up regularly.

## 3. Discussion

Bortezomib is a proteasome inhibitor which is US Food and Drug Administration (FDA) approved for treatment of multiple myeloma and mantle cell lymphoma. Shortt et al. reported the first case of remission of refractory TTP following administration of one cycle of bortezomib [6]. Since then a few other reports have emerged demonstrating similar success [7–12]. A review article on bortezomib therapy for relapsed/refractory TTP nicely summarizes these cases [13]. Differences in dose, schedule, and administration routes of bortezomib in the reported cases have also been covered in that review [13]. A noteworthy point is the difficulty in establishing the efficacy of bortezomib in these cases. van Balen et al., for example, used one cycle of bortezomib to treat refractory TTP in a 16-year-old girl. However, they could not demonstrate if the response was secondary to rituximab or bortezomib [7]. Similarly, Patriquin et al. reported a series of six refractory TTP patients all of whom received PEX, methylprednisolone, and rituximab besides bortezomib [11]. Bortezomib was given very early in the disease process (range 6–22 days) which is understandable given the often aggressive nature of TTP and hence the resultant desperate therapy alterations. Unfortunately though, separating the effects of other therapies from bortezomib becomes difficult and leads to ambiguity of its true efficacy in those patients. Only four other cases of refractory TTP that responded to two or more bortezomib cycles exist in the literature to the best of our knowledge [8–10, 12]. Our case adds to this very scant existent literature.

Amongst patients with ADAMTS13 activity <10%, an ADAMTS13 inhibitor titer of >2 IU/mL has been demonstrated to correlate with significantly lower survival [14]. Indeed, our patient had persistently high inhibitor titer levels (>8 IU/mL) suggesting a more aggressive disease course. The decrease in ADAMTS13 inhibitor titer was maintained only after cycle 3 of bortezomib therapy. Unlike a previously reported case of TTP that did not respond to subcutaneous (cycle 1) bortezomib but responded to intravenous (cycle 2) bortezomib, our case had the opposite finding [10]. We surmise that the route of administration does not play a major role. Rather, it is likely that the total number of bortezomib doses required differs between patients. Subcutaneous and intravenous administration led to equivalent bortezomib plasma exposure and blood 20S proteasome inhibition effects in one study [15]. Further, subcutaneous and intravenous administrations of bortezomib have been demonstrated to have comparable outcomes in relapsed/refractory multiple myeloma [16]. However, peripheral neuropathy incidence is significantly lower with subcutaneous versus intravenous bortezomib [16].

The mechanism of action of bortezomib in TTP is thought to be inhibition of autoantibody generation by inducing apoptosis in both B-cells and plasma cells [6]. This is important because rituximab, an anti-CD20 antibody, destroys only B-cells which express CD20 marker but not the plasma cells. The surviving plasma cells, after rituximab, could serve as a source of autoantibody production which keeps TTP active. Indeed, depletion of circulating CD19/20 positive B-cells by flow cytometry has been shown to occur in patients receiving rituximab for TTP [17]. At least two case reports have demonstrated similar effective CD20 B-cell depletion by flow cytometry (one from marrow aspirate) prior to bortezomib administration [6, 8]. The hypothesized mechanistic role may argue for early usage of bortezomib rather than necessarily after failure of rituximab or that of other therapies in the treatment course of refractory TTP. However, adverse event profile and cost analysis should drive the selection process at least until additional data is available. It is of interest to note that daratumumab, another drug approved for use in multiple myeloma, is a monoclonal antibody against CD38 which is ubiquitously expressed on plasma cells but is also present on regulatory B-cells amongst other immune regulatory cells [18]. Daratumumab was considered for this patient if she remained refractory to bortezomib but has never been used in a TTP patient to our knowledge. Another potential mechanism of action of bortezomib could be its role in inducing apoptosis in immature dendritic cell population which are responsible for processed ADAMTS13 antigen presentation to CD4+ T-cells and thus curbing antibody production [19].

We conclude that bortezomib is an option for treatment of TTP with a sound mechanism of action and an acceptable adverse effect profile. Bortezomib should be considered especially for TTP refractory to PEX, steroids, and rituximab given that the other options in this setting like cyclosporine, cyclophosphamide, vincristine, and splenectomy are supported by limited case series and are accompanied by significant adverse events. While a prospective study of bortezomib in TTP would be ideal, it is unlikely to happen in near future due to the low incidence of relapsed or refractory TTP and the associated difficulty in accrual of patients. As such, retrospective review of cases from large TTP registries will prove important in identifying the role of bortezomib in TTP. Given that publication bias can occur in reporting cases, a large study will help uncover cases where bortezomib failed to induce response in TTP. Overall, it will help define its efficacy, dose, line of therapy, other therapies with which it could be combined, and any role for maintenance. While our case provides one of the longest follow-ups after bortezomib yet, the long-term effects of bortezomib in TTP also need more elucidation. For now, carefully selected cases of refractory TTP should be treated with bortezomib in a manner similar to its incorporation in multiple myeloma regimens. Patients with even severe, relapsed, and refractory TTP like our patient can be safely and successfully treated with multiple cycles of bortezomib.

## References

[1] Reese J. A., Muthurajah D. S., Hovinga J. A. K., Vesely S. K., Terrell D. R., George J. N. (2013). Children and adults with thrombotic thrombocytopenic purpura associated with severe, acquired Adamts13 deficiency: comparison of incidence, demographic and clinical features. *Pediatric Blood and Cancer*.

[2] Sayani F. A., Abrams C. S. (2015). How I treat refractory thrombotic thrombocytopenic purpura. *Blood*.

[3] Scully M., Cohen H., Cavenagh J. (2007). Remission in acute refractory and relapsing thrombotic thrombocytopenic purpura following rituximab is associated with a reduction in IgG antibodies to ADAMTS-13. *British Journal of Haematology*.

[4] Rubia J. D. L., Moscardó F., Gómez M. J. (2010). Efficacy and safety of rituximab in adult patients with idiopathic relapsing or refractory thrombotic thrombocytopenic purpura: results of a Spanish multicenter study. *Transfusion and Apheresis Science*.

[5] Froissart A., Buffet M., Veyradier A. (2012). Efficacy and safety of first-line rituximab in severe, acquired thrombotic thrombocytopenic purpura with a suboptimal response to plasma exchange. Experience of the French Thrombotic Microangiopathies Reference Center. *Critical Care Medicine*.

[6] Shortt J., Oh D. H., Opat S. S. (2013). ADAMTS13 antibody depletion by bortezomib in thrombotic thrombocytopenic purpura. *New England Journal of Medicine*.

[7] van Balen T., Schreuder M. F., de Jong H., Van de Kar N. C. A. J. (2014). Refractory thrombotic thrombocytopenic purpura in a 16-year-old girl: successful treatment with bortezomib. *European Journal of Haematology*.

[8] Mazepa M. A., Raval J. S., Moll S., Ma A., Park Y. A. (2014). Bortezomib induces clinical remission and reduction of ADAMTS13 inhibitory antibodies in relapsed refractory idiopathic thrombotic thrombocytopenic purpura. *British Journal of Haematology*.

[9] Yates S., Matevosyan K., Rutherford C., Shen Y.-M., Sarode R. (2014). Bortezomib for chronic relapsing thrombotic thrombocytopenic purpura: a case report. *Transfusion*.

[10] Patel P. P., Becker J., Freyer C., Griffiths E., Thompson J. E., Wang E. S. (2016). Rituximab-refractory thrombotic thrombocytopenic purpura responsive to intravenous but not subcutaneous bortezomib. *Transfusion*.

[11] Patriquin C. J., Thomas M. R., Dutt T. (2016). Bortezomib in the treatment of refractory thrombotic thrombocytopenic purpura. *British Journal of Haematology*.

[12] Acedillo R. R., Govind M., Kashgary A., Clark W. F. (2016). Treatment of severe, refractory and rapidly evolving thrombotic thrombocytopenic purpura. *BMJ Case Reports*.

[13] Eskazan A. E. (2016). Bortezomib therapy in patients with relapsed/refractory acquired thrombotic thrombocytopenic purpura. *Annals of Hematology*.

[14] Kremer Hovinga J. A., Vesely S. K., Terrell D. R., Lammle B., George J. N. (2010). Survival and relapse in patients with thrombotic thrombocytopenic purpura. *Blood*.

[15] Moreau P., Karamanesht I. I., Domnikova N. (2012). Pharmacokinetic, pharmacodynamic and covariate analysis of subcutaneous versus intravenous administration of bortezomib in patients with relapsed multiple myeloma. *Clinical Pharmacokinetics*.

[16] Arnulf B., Pylypenko H., Grosicki S. (2012). Updated survival analysis of a randomized phase III study of subcutaneous versus intravenous bortezomib in patients with relapsed multiple myeloma. *Haematologica*.

[17] Scully M., McDonald V., Cavenagh J. (2011). A phase 2 study of the safety and efficacy of rituximab with plasma exchange in acute acquired thrombotic thrombocytopenic purpura. *Blood*.

[18] Krejcik J., Casneuf T., Nijhof I. S. (2016). Daratumumab depletes CD38+ immune regulatory cells, promotes T-cell expansion, and skews T-cell repertoire in multiple myeloma. *Blood*.

[19] Park S. J., Cheong H. I., Shin J. I. (2013). Antibody depletion by bortezomib through blocking of antigen presentation. *New England Journal of Medicine*.

